# GPs’ use of gut feelings when assessing cancer risk: a qualitative study in UK primary care

**DOI:** 10.3399/bjgp21X714269

**Published:** 2021-03-23

**Authors:** Claire Friedemann Smith, Benedikte Møller Kristensen, Rikke Sand Andersen, FD Richard Hobbs, Sue Ziebland, Brian D Nicholson

**Affiliations:** Nuffield Department of Primary Care Health Sciences, University of Oxford, Oxford, UK.; Research Unit for General Practice, Department of Public Health, Aarhus University and University of Copenhagen, Denmark.; Institute for Public Health — General Practice, University of Southern Denmark, Odense, Denmark.; Nuffield Department of Primary Care Health Sciences, University of Oxford, Oxford, UK.; Nuffield Department of Primary Care Health Sciences, University of Oxford, Oxford, UK.; Nuffield Department of Primary Care Health Sciences, University of Oxford, Oxford, UK.

**Keywords:** cancer, clinical decision making, clinical guidelines, referral and consultation

## Abstract

**Background:**

The use of gut feelings to guide clinical decision making in primary care has been frequently described but is not considered a legitimate reason for cancer referral.

**Aim:**

To explore the role that gut feeling plays in clinical decision making in primary care.

**Design and setting:**

Qualitative interview study with 19 GPs in Oxfordshire, UK.

**Method:**

GPs who had referred patients to a cancer pathway based on a gut feeling as a referral criterion were invited to participate. Interviews were conducted between November 2019 and January 2020, and transcripts were analysed using the one sheet of paper method.

**Results:**

Gut feeling was seen as an essential part of decision making that facilitated appropriate and timely care. GPs distanced their gut feelings from descriptions that could be seen as unscientific, describing successful use as reliant on experience and clinical knowledge. This was especially true for patients who fell within a ‘grey area’ where clinical guidelines did not match the GP’s assessment of cancer risk, either because the guidance inadequately represented or did not include the patient’s presentation. GPs sought to legitimise their gut feelings by gathering objective clinical evidence, careful examination of referral procedures, and consultation with colleagues.

**Conclusion:**

GPs described their gut feelings as important to decision making in primary care and a necessary addition to clinical guidance. The steps taken to legitimise their gut feelings matched that expected in good clinical practice.

## INTRODUCTION

‘Gut feelings’ guide clinical decision making, can prompt investigation for a range of conditions including cancer,^1^^–^^4^ and are conceptualised as a rapid summing up of multiple verbal and non-verbal cues in the context of a GP ’s knowledge and experience.^3^^,^^5^ GPs have often struggled to articulate their experience of a gut feeling, referring to them as alarm bells sounding, or a physical sensation like the hairs on the back of their neck rising or a lurch in their stomach.^6^^,^^7^ Erik Stolper and colleagues have established a common dialect for gut feelings across countries^5^^,^^8^^–^^10^ and languages,^11^ with two types of gut feelings commonly described: a sense of alarm or an uneasy feeling; or a feeling of reassurance or confidence about the health of the patient.^12^

Research into gut feelings and their use in primary care has increased over recent decades.^3^ During this period there has also been an increase in demand for primary care,^13^ wide variation in GPs’ access to cancer investigations,^14^ and losses in continuity of care^15^ that may damage the doctor–patient relationship regarded as the cornerstone of primary care practice.^16^ Additionally, in some countries urgent referral pathways have been developed to improve outcomes in patients with ‘red-flag’ cancer symptoms.^17^^–^^19^ These pathways have reduced variation in clinical practice and are associated with reduced cancer-related mortality,^20^ but may have disadvantaged patients with cancer who present with non-specific symptoms.^21^^–^^23^ Interest in gut feelings has grown from accounts of their predictive value for cancer in patients with non-specific symptoms, and they have been included in some referral guidelines.^3^ However, gut feelings remain a controversial referral criterion, critiqued as subjective and contrary to evidence-based medicine (EBM).^24^^–^^26^

The present study aimed to explore the role that gut feeling plays in primary care clinical decision making through discussions with GPs who had recently referred to a cancer pathway based on gut feeling.

## METHOD

### Recruitment

GPs who had referred a patient to the Suspected Cancer (SCAN) Pathway, a referral pathway for patients with nonspecific symptoms of cancer operating in Oxfordshire, UK, which includes *‘GP clinical suspicion of cancer or serious disease/GP gut feeling’* as a referral criterion,^27^ were eligible. GPs who had referred at least one patient within 1 year of recruitment initiation (October 2018 to October 2019), and were still working at the practice from which they had made their referral, were identified from the SCAN Pathway database and contacted via email. GPs were contacted irrespective of whether they had made a referral based on a gut feeling. Recruitment emails included a study introduction and a participant information sheet explaining the study’s focus on gut feelings. GPs were requested to contact the study team if they had any questions or wished to take part.

**Table:** How this fits in

GPs’ gut feelings have often been criticised because of their subjective nature. GPs suggested that they did not rely on gut feelings in isolation but used them as prompts to gather additional clinical evidence to support their decisions, and to reduce the potential criticism of being ‘unscientific’. They stated that gut feelings were integral to efficient and professional patient care, particularly when the presentation causing concern fell into a ‘grey area’ of clinical practice that guidelines do not adequately address. As gut feelings were described as most reliable when used by an experienced GP, grounded on years of observations and accumulated clinical knowledge, there may be a role for mentoring less experienced GPs to understand and respond appropriately to gut feelings.

### Patient and public involvement

A focus group was held in November 2018 with five patients to gain insight into the interest and relevance of GPs’ and patients’ gut feelings for cancer and other serious illness to patients, and gather feedback on the draft interview schedule.

### Sample

Interviews were conducted with the 19 GPs who responded to the invitation to participate. The participating GPs had been qualified for between 1 and 30 years; 11 were female; 10 were salaried GPs; and nine were partners in their practices. Though GPs who had not cited gut feeling as a reason for referral were contacted, none of these GPs responded to the invitation to participate.

### Interviews

Interviews, lasting an average of 59 minutes (range 47–73 minutes), were conducted by one of two authors (both anthropologists), either face to face or over the telephone between November 2019 and January 2020. The semi-structured interview schedules were informed by the authors’ recent systematic review of the gut feeling literature^3^ and the patient and public involvement (PPI) group. Written informed consent was obtained at the start of the interview and GPs were given an opportunity to ask questions. The signed consent form was returned to the research team before the telephone interviews took place and consent was confirmed before the interview began. Interviews began with a discussion of the GP’s career before considering gut feelings in general and in relation to cancer suspicion specifically, with the circumstances leading to the GP’s referral to the SCAN Pathway used as case studies.

### Analysis

Interviews were digitally recorded and transcribed verbatim. Transcripts were coded using NVivo (version 12) into anticipated and emergent themes. These were discussed and elaborated with reference to existing literature, by four of the authors, using a mind mapping ‘one sheet of paper’ (OSOP) method.^28^ In the OSOP method, issues arising within codes are noted along with the contributing participant identification numbers. Once a summary of all the issues had been produced, all of the authors considered how these fitted together to form the narrative of each theme.

## RESULTS

### Gut feelings and GP experience

GPs often struggled to verbalise what gut feelings are and how they are used, but also positioned them as integral to efficient and professional care with the potential to change the ‘route’ of the GP’s thinking:

*‘*[Gut feeling] *will make me think, ah, that’s not normal. You know, that’s not the right way, that we should be going down on this route, there’s something else going on here.’*

(GP12, female [F], 2-years qualified)

Accounts suggested that those expressing some discomfort with using gut feelings as a referral criterion were concerned that they might be seen as ‘unscientific’. Some of the GPs worked hard to distance the decision making they attributed to gut feelings from concepts such as ‘instinct’, being ‘magical’, or a ‘sixth sense’ (GP06, F, 8-years qualified):

*‘I don’t want to say instinct cos* [ *sic*] *it’s so unscientific, but* [gut feeling is] *that feeling that something is amiss, and isn’t explained by what you have so far*. *’*

(GP02, F, 6-years qualified)

By presenting gut feelings as grounded in clinical knowledge, GPs challenged the notion that gut feelings are ‘unscientific’ and emphasised the importance of amassing broad clinical experience before gut feelings could be considered reliable:

*‘… the more and more exposures you have to similar cases and different cases, the more basis you have for your gut feeling. And the more informed it is, so I suspect that sort of more experienced clinicians’ gut feeling is more refined than more junior clinicians*’ *.’*

(GP10, F, 1-year qualified)

Some GPs contrasted their current experience with earlier stages of their career or training, expressing their growing confidence in making decisions based on their gut feelings. Some of the more recently qualified GPs also anticipated that, like a skill, their gut feelings would become more accurate and their confidence would grow with increasing experience and use:

‘… my impression is that one becomes more trusting of one’s gut feeling as you get more experienced, I think.’

(GP09, Male [M], 4-years qualified)

Many of the GPs described developing confidence in their own gut feelings as punctuated by cases that had changed the way they thought about a set of symptoms. These cases often involved unusual presentations, or missed or delayed diagnoses, that made a lasting impression, as one GP expressed:

‘We make good decisions because we’ve made bad decisions.’

(GP01, M, 25-years qualified)

As well as experience, GPs suggested that successfully using gut feelings required an ability to recognise patterns supported by well-developed observational skills, and that gut feelings were the result of a multitude of observations that are often so subtle they are imperceptible to a bystander:

‘… all of these little things we’re kind of reading and drawing on all the time, and they’re all adding to us being able to make an impression and sometimes, though, the impression is very obvious and easy to describe, but sometimes it’s more nebulous and there’s just something that just doesn’t sit right with the patient.’

(GP09, M, 4-years qualified)

Additionally, some of the more recently qualified GPs suggested that personality was a factor in using gut feelings as it requires empathy, an ability to recognise gut feelings, and a willingness to use them:

*‘I think whether people recognise it consciously or subconsciously probably changes* […] *I think people are more or less aware of it, more or less happy to use it.’*

(GP10, F, 1-year qualified)

*‘I think it’s* [gut feeling] *having the interest in patients, and having the empathy for patients, I think it’s quite easy to ignore your gut feelings and just go, “Do this, do this, do this, off you go, fine.” And sort of treat things very clinically.’*

(GP14, F, 2-years qualified)

These additional factors meant that successful use of gut feelings was positioned as partly owing to learnt skills, the culture in which GPs trained and practised, and personal ways of practising, and not just owing to clinical experience *.* The desirable characteristics of empathy and self-awareness were also put forward as an argument for gut feeling use by some GPs, sometimes to the extent that a good GP was one who used gut feelings effectively:

‘… perhaps some doctors don’t have that, you know ability to draw on that gut feeling, because they might not be you know, quite so good, just in general you know as, as a doctor.’

(GP12, F, 2-years qualified)

### Using gut feelings to navigate the ‘grey area’

Many GPs suggested that there were aspects of primary care that made gut feeling use necessary. In particular, several stated that they were uniquely placed to understand their patient as a whole person, and that the role of the GP was to go beyond formalised medical knowledge, as GP10 explained:

*‘… in the end a lot of what we do, gut feeling contributes* […] *we’re not just guideline machines, there to gate keep access to services* […] *Our job is to look at the whole person and work out with them what they’re worried about, what we’re worried about, yes, what the evidence says, and what guidelines would say* […] *People are more than just a list of binary yes or no tick boxes.’*

(GP10, F, 1-year qualified)

These GPs recognised that: *‘referral criteria* [are] *deliberately non-specific and deliberately don’t exclude very many patients, there’s quite a big grey area’* (GP17, M, 9-years qualified) and that it is in this grey area that using clinical judgement is vital to interpret clinical guidance. Patients were described as falling into the grey area if guidance was inadequate for their presentation. This could be because the patient did not present with symptoms that were included as referral criteria in the guidelines but the GP assessed the patient to be at risk of cancer:

*‘… no one of these individual lab findings would have triggered a 2-week wait pathway referral for any one system. And each of them by themselves I could have explained* […] *but put all together, they were all just little warning flags that made me feel uncomfortable* […] *Previously they’d been very independent, and that to me was a bit of a, red flag, and that triggered that gut feeling of I worry that there’s something else here, that could be related to their cancer. And unfortunately it was.’*

(GP10, F, 1-year qualified)

Alternatively, a patient could fall into the grey area if the GP judged their presentation to be within what could be considered normal for them despite it being an indication for referral in the guidelines, an increasingly common scenario with the widening of referral criteria to include nonspecific symptoms:

*‘… we have guidelines, pathways, and everything else, and somebody who comes in coughing up blood gets that, that, and that* […] *But three people who come in and have been coughing up blood come with three different scenarios and three different backgrounds and might actually, for one of them it might be quite normal, because they’ve been coughing up blood for a long time.’*

(GP15, M, 25-years qualified)

As such, the grey area was described as being located between what is normal and what is abnormal, and required contextual knowledge and interpretation by the GP as well as a level of vigilance that they would associate with using their gut feelings. Gut feelings were described as coming to the fore in *‘a grey area where there’s no rules that fit what you’re faced with so you fall back on your experience’* (GP03, F, 9-years qualified) in order to catch patients that guidelines would miss. Some GPs stated that the more relational aspects of primary care, such as their knowledge of their patient, was too nuanced and patient specific to be included in guidance, but no less legitimate for decision making:

*‘I think what we mean by gut feeling is,* […] *we’re drawing on physical signs, and little subtle features of the patient’s behaviour* […] *all of these things are just drawing on information all the time* […] *And I think it has a role.’*

(GP09, M, 4-years qualified)

The authors suggest a definition for ‘the grey area’ in Box 1, built on the narratives of the GPs interviewed herein and the work of earlier researchers.^29^

**Box 1.** The grey area

Modern medicine has blurred the distinction between health and illness through the inclusion of increasingly vague and non-specific bodily experiences as potential markers of disease and drives towards earlier diagnosis of disease. General practice incorporates longitudinal care that allows an in-depth knowledge of the patient to be built up over time. Located in the overlap between what is considered normal and abnormal, the grey area requires the GP to be vigilant and to interpret the clinical scenario in the context of what they know about the patient personally and other people like them. The grey area represents the dissonance between accepted wisdom about which signs and symptoms represent significant illness and what the GP knows about the health of their patient, which can result in a gut feeling. A patient falls into the grey area when the GP assesses them to be at risk of serious illness despite their symptoms not being included as referral criteria in guidelines. The patient may also fall into the grey area if the GP judges the patient’s presentation to be within what could be considered normal *for them* despite the clinical presentation being an indication for referral in the guidelines. Under both circumstances the GP may feel compelled to act in a way not supported by clinical guidance, but supported by their gut feeling.

### Building a case for decisions based on gut feelings

Several strategies were described to bolster gut feelings, primarily so that GPs’ decisions or requests for further investigations would be accepted and to avoid *‘being led up the garden path’* (GP09, M, 4-years qualified). Strategies included building an evidence base through further questioning about symptoms and examining referral guidelines to see how the patient could be fitted to the criteria. Supporting evidence was also sometimes sought by ordering additional tests if the test results on record were unrelated to the current clinical presentation, or if additional tests were required for the patient to qualify for referral:

*‘… we will attempt to put it* [gut feeling] *in some kind of framework that we think will be recognisable to a specialist nurse or a junior doctor, who’s reading the referral in clinic, because we don’t want the referral to be dismissed* […] *We want people to take it seriously.’*

(GP17, M, 9-years qualified)

The second opinion of GP colleagues, particularly those more experienced GPs, was described as a useful source of validation of gut feelings. Many of the GPs said that they discussed gut feelings with their colleagues and in doing so were able to drill down to the gut feeling contributors, and sometimes provoked a similar feeling in the colleague:

*‘… we also use it* [referrals meeting] *to discuss difficult or complex cases, or cases where we just have that gut feeling of, “I’m uncomfortable with this, and I need to explore it more*” […] *It makes you identify the key features that you’re feeling uncomfortable with and describe them to someone else, and you can often get a reaction from refining just those things, you can get a reaction of them saying, “Phh, my gut feeling says that’s nothing”*, *or, “Ooh, my gut feeling is saying actually, those add up to alarm symptoms.”’*

(GP10, F, 1-year qualified)

### Acting on gut feelings

Descriptions of actions that would be taken in response to a gut feeling frequently mirrored descriptions of what was considered good practice. These actions included redoubled efforts and increased urgency to uncover what was causing the patient’s symptoms, as well as advocating or even ‘fighting’ for access to investigations in secondary care:

‘… sometimes you feel like you’re really advocating for your patients, and you, you are concerned about your gut feelings and you’re fighting to get them seen.’

(GP14, F, 2-years qualified)

Some GPs said that using their gut feeling to negotiate investigations to rule out disease could also be useful, acknowledging that some patients for whom they had experienced a gut feeling were not diagnosed with cancer, but that this in itself could be valuable. None of the GPs described instances of an incorrect gut feeling for cancer that they felt had been harmful. This was qualified by many who stressed that, while this was the case, it was still necessary to avoid overburdening the system, causing the patient anxiety, and to be *‘mindful of not over-investigating people’* (GP09, M, 4-years qualified), ensuring that, if gut feeling is used, a thorough assessment of the patient is still carried out.

Several GPs said that it was unlikely they would ignore a gut feeling. The few examples of when they would act counter to gut feeling were when the gut feeling was reassuring. In this instance they said that, despite their gut feeling, they might still order some tests as the consequences of missing a diagnosis were worse than those of investigating the patient unnecessarily:

‘I feel, my confidence grows in being able to listen to gut feelings that tell me, “Look there’s nothing going on here”, you don’t need to investigate them to the, you know nth degree. You can do what seems sensible, and if those things are normal, you, there is nothing going on here.’

(GP14, F, 2-years qualified)

GP14 and several others described how primary care is becoming increasingly risk averse and litigious, with investigations often being the only way to provide patients with adequate reassurance. As such, they had become ‘fearful’ of receiving a complaint and so more inclined to practise defensively:

*‘I think my level of tendency to investigate people is probably a bit higher now than it used to be, which is ironic, because I’m more experienced. So, you might think that it had gone the other way, but I am fearful about you know a complaint or so forth* […] *It’s hard to look at somebody and say, “Ah you look really well, so there’s nothing wrong with you.”’*

(GP08, F, 30-years qualified)

### Gut feelings and the GP’s professional role

When GPs felt the need to make a clinical decision based on a gut feeling, they often described having discussions directly with the secondary care colleagues to whom they were hoping to refer their patients. Many of the descriptions of this interaction resulted in the consultant agreeing to see the patient or suggesting a more appropriate referral route. Success stories of using this strategy tended to be told by GPs with greater experience:

*‘If I say to a more senior surgeon or physician, this patient’s not well and I’m just not happy managing them in the community, in a way it doesn’t matter what the parameters are* […] *if I’m not happy then they’re not happy either, and will take it.’*

(GP11, F, 26-years qualified)

*‘I have referred a few people in like that* [on a gut feeling] *before, and I’ve not had the best response* […] *I might be being over simplistic but, I would never write, “I’ve got a gut feeling” on a referral letter.”’*

(GP12, F, 2-years qualified)

Frequent comparisons were made between primary and secondary care practice, and the GPs often concluded that the use of gut feelings in clinical decision making was necessary and sensible, and set primary care apart from secondary care. Furthermore, the acceptance of GPs’ gut feelings by secondary care colleagues signified recognition of the GP’s expertise and in-depth knowledge of their patients:

*‘Actually I think it shows a professional respect between secondary care and primary care* […] *to say, “We recognise that you know your patient and you are worried.”’*

(GP10, F, 1-year qualified)

## DISCUSSION

### Summary

Participants in this study distanced themselves from notions of gut feelings being ‘magical’ or ‘unscientific’, and instead emphasised that gut feelings were a marker of good clinical practice based on experience and contextual knowledge, attuned over years of observation, and a legitimate basis for clinical decision making. The ability to make a decision based on a gut feeling was especially important when the clinical scenario fell into the referral grey area, which has become a feature of primary care, and the GP’s assessment of cancer risk was at odds with clinical guideline recommendations. GPs acknowledged that the subjective nature of gut feelings could possibly lead to over-investigation and patient anxiety. As such, gut feelings were used to prompt further clinical enquiry, investigation, or referral, and sometimes to gain a second opinion of a (ideally more experienced) colleague. A secondary care clinician’s acceptance of a GP’s gut feeling was considered a marker of professional respect.

The grey area was seen as a range of clinical presentations that fell across what is considered normal and disease signalling, where experience, contextual knowledge, and vigilance on the part of the GP were required to ensure the patient was cared for appropriately. Effective use of gut feelings may be facilitated by focusing on how GPs should use gut feelings, communicate their suspicions to colleagues, and by finding ways to share experienced GPs’ wisdom with their newly trained and qualified colleagues.

### Strengths and limitations

A strength of this study is that it discusses practicalities of incorporating gut feelings into the clinical decision making of GPs who have recent experience of using gut feeling as a reason for referral. The present GP sample was varied in terms of time spent in practice and experiences before qualifying, and as such provides a description of gut feeling use from a range of viewpoints.

The study also has some limitations. All the GPs interviewed had made a referral to the SCAN Pathway based, at least partially, on a gut feeling and could be viewed as atypical. The authors would expect GPs who believed that they did not use gut feelings in their practice to have a different view of the role, if any, that gut feeling plays in decision making, and this perspective is missing from the interviews. GPs who had not used gut feeling as a reason for referral may have felt that the research topic was less relevant to them, and the link between willingness to participate in research and perceived relevance has been noted previously.^30^ As discussed earlier, the ability of GPs to discuss gut feelings with the benefit of recent relevant experience adds a unique, if narrower, viewpoint to the literature and may also be seen as a strength. Additionally, interviews were conducted up to 1 year after the referral was made that lead to the invitation to participate. As such, the GPs may not have remembered the circumstances of the referral clearly. However, while the discussion of gut feeling used the referral as a way to begin the conversation, the majority of the interviews were about the use of gut feeling generally, so the authors do not believe that this limitation would have influenced the present findings substantially.

### Comparison with existing literature

Gut feelings have previously been described as a prompt to search for objective evidence.^7^^,^^24^^,^^31^ For the present study participants, gut feelings functioned as a prompt to initiate investigations and engage in diagnostic reasoning for patients who can have difficulty accessing established routes to further care because of the non-specific nature of their presentation.^32^

Participants in the present study echoed the findings of previous studies that gut feelings were grounded in longitudinal relationships, with patients giving the GP the ability to notice changes from what is normal for individual patients.^3^^,^^5^ This was particularly important for the concept of a ‘grey area’ in clinical decision making, which emerged during these interviews as the main area in which gut feelings were used. The authors defined ‘grey area’ (Box 1) by building on the narratives of the present study participants and the work of earlier researchers.^29^ While the challenge of investigating patients whose presentation does not fit referral guidelines has been discussed previously,^33^^,^^34^ the present GP participants described the grey area as not only where the patient’s presentation is not covered by guidance but also where guidelines do not provide enough distinction between normal and abnormal. As such, navigating this overlap between the normal and abnormal draws on the GP’s relationship with their patient and thus their ability to apply their contextual knowledge to interpret the patient’s presentation.^35^

### Implications for research and practice

The GPs interviewed were clear that ‘gut feelings’ are an important part of ‘clinical judgement’ and the terms are often used interchangeably in the literature.^3^ Bodies such as the National Institute for Health and Care Excellence (NICE) incorporate statements that clinical guidance should not override clinical judgement.^25^ Examples of the difficulties GPs face justifying action contrary to clinical guidance can be found both in the literature^3^ and in the present analysis. The concern that gut feeling-based referral criteria would be used irresponsibly is shared even by GPs who support their use. The authors suggest that clinical guidelines could outline the ways in which GPs might act on gut feelings for patients that fall into the grey area. Using gut feelings to prompt more detailed enquiry, closer examination of the patient, and seeking the input of colleagues seems uncontroversial. Detailed record keeping of these actions and referral forms that include the opportunity to provide a clinical narrative would support this. The most effective way for GPs to communicate their gut feelings to clinicians triaging referrals should be explored further.

GPs draw a strong connection between clinical experience and reliable gut feelings. However, restricting the use of gut feelings to those deemed experienced ‘enough’ presents a variety of problems including determining what ‘enough’ experience is, especially as good observation skills and empathy were also considered important determinants of reliable gut feelings, traits not so closely tethered to the amount of clinical experience. Case studies or mentorship schemes, for example, could provide opportunities for experienced GPs to share insights of when and how to safely incorporate gut feelings into clinical decision making.
